# Leakage Detection in Subway Tunnels Using 3D Point Cloud Data: Integrating Intensity and Geometric Features with XGBoost Classifier

**DOI:** 10.3390/s25144475

**Published:** 2025-07-18

**Authors:** Anyin Zhang, Junjun Huang, Zexin Sun, Juju Duan, Yuanai Zhang, Yueqian Shen

**Affiliations:** 1 Jiangsu Geological Engineering Survey Institute, Nanjing 210018, China; zhanganyinjs@163.com (A.Z.); sun_zexin@163.com (Z.S.); njutdjj@163.com (J.D.); 2 School of Earth Sciences and Engineering, Hohai University, Nanjing 211100, China; 3 School of Geography, Nanjing Normal University, Nanjing 210023, China; 18795895163@163.com

**Keywords:** point cloud, leakage detection, intensity features, geometric features, XGBoost

## Abstract

Detecting leakage using a point cloud acquired by mobile laser scanning (MLS) presents significant challenges, particularly from within three-dimensional space. These challenges primarily arise from the prevalence of noise in tunnel point clouds and the difficulty in accurately capturing the three-dimensional morphological characteristics of leakage patterns. To address these limitations, this study proposes a classification method based on XGBoost classifier, integrating both intensity and geometric features. The proposed methodology comprises the following steps: First, a RANSAC algorithm is employed to filter out noise from tunnel objects, such as facilities, tracks, and bolt holes, which exhibit intensity values similar to leakage. Next, intensity features are extracted to facilitate the initial separation of leakage regions from the tunnel lining. Subsequently, geometric features derived from the k neighborhood are incorporated to complement the intensity features, enabling more effective segmentation of leakage from the lining structures. The optimal neighborhood scale is determined by selecting the scale that yields the highest F_1-score_ for leakage across various multiple evaluated scales. Finally, the XGBoost classifier is applied to the binary classification to distinguish leakage from tunnel lining. Experimental results demonstrate that the integration of geometric features significantly enhances leakage detection accuracy, achieving an F_1-score_ of 91.18% and 97.84% on two evaluated datasets, respectively. The consistent performance across four heterogeneous datasets indicates the robust generalization capability of the proposed methodology. Comparative analysis further shows that XGBoost outperforms other classifiers, such as Random Forest, AdaBoost, LightGBM, and CatBoost, in terms of balance of accuracy and computational efficiency. Moreover, compared to deep learning models, including PointNet, PointNet++, and DGCNN, the proposed method demonstrates superior performance in both detection accuracy and computational efficiency.

## 1. Introduction

Rail transit constitutes a critical component of urban infrastructure, influencing the development and spatial organization of contemporary cities. With the rapid expansion of subway networks, tunnel systems experience numerous structural challenges, including water leakage [1], cracks [2], segment misalignment [3], cross-sectional deformation [4], and uneven settlement [5]. Among these issues, water leakage emerges as the most prevalent and consequential structural damage. This phenomenon accelerates weathering, lining spalling, and corrosion of tunnel lining, compromising structural integrity and posing significant threats to operational safety. Consequently, regular and effective inspection for tunnel leakage represents a critical priority for subway system maintenance [6]. Traditional inspection methods typically rely on manual photography, and entail a high level of subjectivity as well as the safety hazards inherent in confined and complex tunnel environments. These methods are widely criticized for their inefficiency, insufficient precision, and occupational risks [7,8].

Recent advancements in mobile laser scanning (MLS) technology have emerged as a promising alternative for tunnel leakage inspection. MLS systems, mounted on a rail trolley, integrate laser scanners, high-definition cameras, odometers, and computing units to automatically acquire high-precision and high-density 3D point cloud data [8]. This technology enables effective 3D reconstruction of tunnel environments and reduces reliance on manual inspection. However, several challenges remain. The irregular shapes and varying scales of leakages complicate detection. Additionally, tunnel point cloud data often contains interference from ancillary facilities, bolt holes, tracks, and noise, which hinder accurate identification. This study addresses these challenges by proposing a novel classification method that combines geometric and intensity features to automatically detect leakage in 3D space. The key contributions of this research are as follows:

(1) An automated framework was developed to accurately detect tunnel leakage in 3D point cloud data, which addresses challenges related to noise and the limited spatial representation of leakage patterns.
(2) Geometric features were introduced as complementary characteristics, which resulted in improved accuracy for leakage detection.
(3) The XGBoost classifier was introduced for leakage detection, and a comparative analysis demonstrated its superior accuracy and computational efficiency, thereby validating its applicability in this context.

The remainder of this study is organized as follows: Section 2 reviews existing studies and summarizes limitations of the literature. Section 3 describes the proposed methodology in detail. Section 4 presents the experimental results and discussion. Finally, Section 5 concludes the study.

## 2. Related Works

Many researchers have explored methods to detect leakage using tunnel point cloud data, which can be broadly categorized into two main approaches:

(1) Intensity threshold-based segmentation methods. These methods convert point clouds into grayscale images by utilizing the intensity values of laser scanning points [7,9,10]. Leakage is detected by establishing intensity thresholds derived from the difference between leakage and non-leakage regions in grayscale domain [11,12]. For example, Huang, et al. [7] employed Otsu’s method to compute grayscale histogram and determine an optimal intensity threshold that maximizes inter-class variance between leakage and non-leakage areas, achieving optimal segmentation results. However, point cloud intensity is susceptible to biases induced by factors such as scanning distance, incidence angle, and surface roughness. Hawley and Gräbe [13] investigated these influences, emphasizing the necessity of intensity correction models to mitigate such deviations [14]. Xu, et al. [15] proposed a two-step approach: first, correcting intensity values based on scanning distance and incidence angle; second, employing an intensity threshold to extract leakage and a distance threshold to eliminate noise points. Despite their effectiveness, these methods exhibit notable limitations. The segmentation process is highly sensitive to the intensity threshold, which is typically determined empirically or heuristically. This approach may not generalize well to diverse datasets, limiting its practical applicability. Furthermore, most implementations are confined to 2D leakage segmentation, lacking the capability to provide detailed 3D information regarding the depth and spatial extent of leakages.
(2) Image-based supervised classification methods. These methods also involve transforming point clouds into grayscale images. Annotated samples are used to train deep learning models, which are then tested on grayscale images to detect leakage locations and areas. Recent advancements in convolutional neural networks (CNNs) have garnered attention for their capability to efficiently extract features. Deep learning frameworks derived from CNNs, such as ResNet [16], Faster R-CNN [17], R-FCN [18], Mask R-CNN [19], DeepLabV3+ [20], and YOLO [21], have been extensively applied to various object detection tasks. These tasks include crack detection [22], defect detection [23], leakage detection [24], etc. For example, Liu, et al. [25] combined Res2Net with cascade modules and fully connected networks (FCNs) to detect leakage, leveraging multi-scale feature extraction and enhanced representation. Among these, Mask R-CNN, one of the most widely used algorithms for leakage instance segmentation, extends Faster R-CNN by integrating Region of Interest (RoI) Align [26] and FCN [27] modules. To enhance the segmentation accuracy, Guo et al. [1] employed RDES-Net to effectively segment leakage on grayscale images. Chen et al. [28] proposed an enhanced YOLO-V7 model that integrates attention mechanisms, edge refinement techniques, and mixed data augmentation strategies to achieve precise leakage segmentation. Wang et al. [29] designed a lightweight leakage segmentation method using the DeepLabV3+ model with integrated channel attention, demonstrating enhanced accuracy and generalization performance in complex environments. To visualize leakage in 3D space, Chen et al. [19] introduced a method to unfold point clouds into grayscale images using cylindrical voxels, enabling Mask R-CNN-based detection and subsequent mapping back to 3D space. Additionally, Xue et al. [30] applied SfM-Deep Learning to map leakage textures onto 3D models, further enhancing spatial analysis capabilities.

A comparative summary of these representative leakage detection methods is presented in Table 1, which details their utilized tunnel structures, data acquisition equipment, input data types, core techniques, reported accuracies, and key limitations. As detailed in Table 1, the image-based supervised classification methods have demonstrated considerable effectiveness, achieving performance metrics such as an IoU exceeding 80% and a max mAP of 76.4%. Despite their promising results, these image-based methods suffer from several fundamental limitations. They primarily rely on intensity features, which overlooks the critical geometric information, especially at regions with distinct geometric deviations like circumferential joints and bolt holes where leakage commonly occurs. Furthermore, as summarized in Table 1, these methods are (1) sensitive to lighting conditions and object occlusion, (2) suffer from a loss of spatial information during 2D projection, and (3) fail to visualize leakage patterns effectively in 3D space.

Additionally, the intensity threshold-based segmentation approaches are also constrained by significant drawbacks. As noted in Table 1, they are (1) sensitive to lighting conditions and object occlusion, (2) highly sensitive to the selection of a varying threshold, and (3) likewise incapable of visualizing leakage in 3D space. These collective limitations highlight the urgent need for a methodology capable of directly detecting leakage from a fully 3D perspective while integrating robust geometric features.

## 3. Materials and Methods

**Figure 1:**
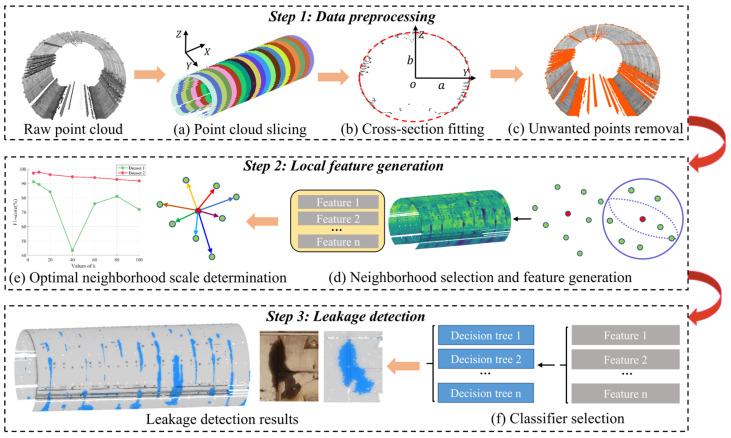
The workflow of the proposed methodology.

This study presents a novel approach for detecting water leakage in shield tunnels using 3D point cloud data. The proposed approach integrates intensity and geometric features with an XGBoost classifier to enhance detection accuracy and efficiency. As illustrated in Figure 1, the methodology comprises three core stages: data preprocessing, local feature generation, and tunnel leakage detection. The preprocessing stage begins with slicing the 3D point cloud data along the longitudinal axis of the tunnel. This slicing facilitates manageable data segmentation, aligning the dataset with the geometric structure of the tunnel. Noise in the point cloud data is then filtered using an enhanced RANSAC algorithm, which incorporates refined random point selection to improve precision of outlier removal. Following noise filtering, samples are selected for training and testing purposes. This step ensures that the dataset is properly balanced and representative of various tunnel leakage scenarios. Post-preprocessing, the framework focuses on extracting relevant features for classification. Geometric features are computed alongside smoothed intensity features, which are generated using a k-nearest neighbors (k-NN) algorithm. The optimal neighborhood scale for feature extraction is determined by analyzing the F_1-score_ across varying k-values. Finally, the XGBoost classifier is employed to integrate these features for leakage detection. XGBoost was adopted for its robustness, scalability, and ability to handle both linear and non-linear relationships in multi-feature datasets. This model effectively differentiates between leakage and non-leakage points, achieving high detection accuracy while maintaining computational efficiency.

### 3.1. Data Preprocessing

#### 3.1.1. Tunnel Slicing Along the Tunnel Axis

**Figure 2:**
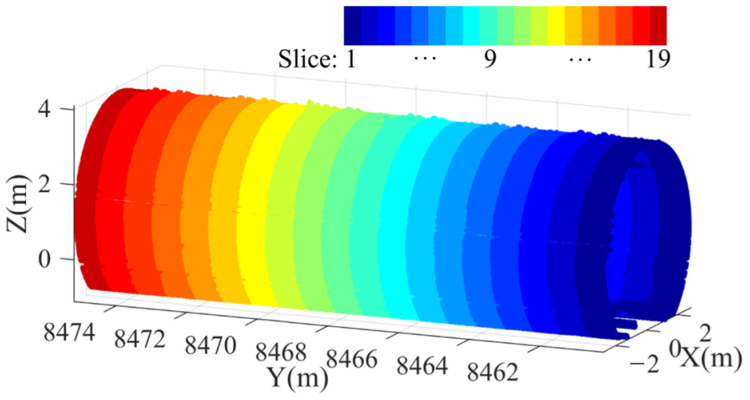
Graphical illustration for tunnel point cloud slicing.

Slicing the tunnel along its longitudinal axis represents a critical preprocessing step for tunnel point cloud data. This segmentation facilitates localized analysis and ensures higher accuracy in subsequent processing tasks. The slicing process begins by identifying the longitudinal axis of the tunnel through principal component analysis (PCA), which extracts the principal axis from the 3D point cloud data to define the slicing direction and maintain geometric alignment [31]. Once the principal axis is established, the point cloud is divided into slices of uniform thickness. Let the point cloud coordinates be $P{\left(x_{i},y_{i},z_{i}\right)}_{i=1,2,\ldots ,n}$, and the slice thickness denoted as w. With the tunnel axis aligned with the *Y*-axis, the number of slices nslice is calculated using Equation (1).

(1) $$nslice=floor\left(\left(y_{\max}-y_{\min}\right)/w\right)$$

where floor(⋅) denotes the ceiling function. The coordinates of the point cloud within each slice are stored as $p_{slice}^{k}$(k=1, 2, …, nslice), enabling spatially segmented analysis. Figure 2 illustrates the slicing process for the tunnel point cloud.

#### 3.1.2. Cross-Section Fitting

After slicing, each point within a slice is projected onto the XOZ plane by ignoring the y-coordinate, resulting in a set of 2D points $p_{slice}{\left(x_{i},z_{i}\right)}_{i=1,2,\ldots ,n}$. To accurately delineate the tunnel’s geometry from these projected points, a process known as cross-section fitting is performed, which involves creating a precise elliptical model to represent the shape of the tunnel slice. For this task, the random sample consensus (RANSAC) algorithm is employed due to its robustness against outliers. The fitting procedure for each cross-section involves the following iterative steps: (1) A minimal subset of points is randomly selected from the projected data. (2) An ellipse is fitted using selected subset. (3) All other points in the cross-section are tested against this ellipse, and the number of points (inliers) that fit the model within a predefined tolerance is counted. These steps are repeated for a set number of iterations, and the ellipse with the largest set of inliers is selected as the final, robust representation of the tunnel’s cross-section.

To enhance robustness of ellipse fitting, points corresponding to tracks and ground are excluded during the random sampling process, as these irrelevant or outlier points are geometrically distant from the tunnel lining. Including these irrelevant points in the process of fitting could distort the ellipse fitting and compromise the accuracy of cross-sectional geometry extraction. A polar coordinate system is introduced to streamline the process. The mean coordinates $\left(\overset{-}{x},\overset{-}{z}\right)$ of all points in each slice are calculated and serve as the origin of the polar coordinate system. For each point, polar coordinates $\left(r_{i},\theta_{i}\right)$ relative to this origin $\left(\overset{-}{x},\overset{-}{z}\right)$ are derived using Equation (2).

(2) $$\left\{\begin{array}{l} r_{i}=\sqrt{{\left(x_{i}-\overset{-}{x}\right)}^{2}+{\left(z_{i}-\overset{-}{z}\right)}^{2}}\\ \theta_{i}=\arctan2\left(z_{i}-\overset{-}{z},x_{i}-\overset{-}{x}\right) \end{array}\right.$$

**Figure 3:**
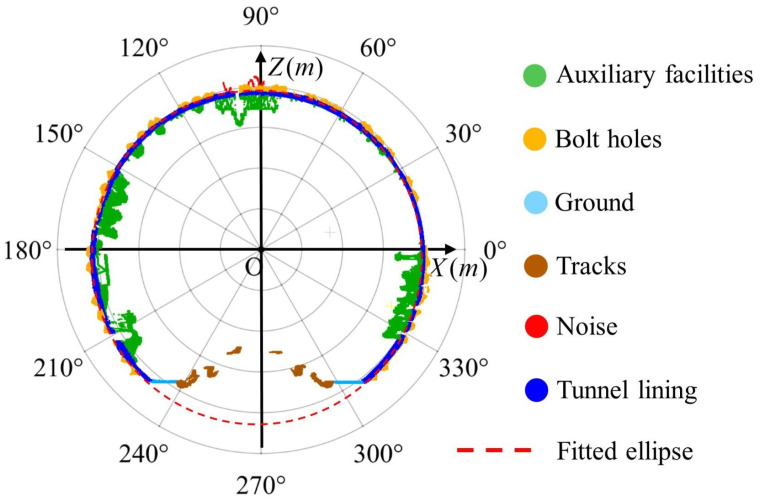
Schematic diagram of tunnel cross-section point cloud for polar coordinate calculation.

This polar representation enables angular filtering of points to isolate track and ground regions. Typically, these points fall within angular ranges of approximately 230 degrees to 310 degrees in cross-sectional polar coordinates, as shown in Figure 3. The ellipse is fitted using the RANSAC algorithm on points outside these angular ranges, and its geometry is described by the standard ellipse equation, as shown in Equation (3).

(3) $$\frac{{\left(x-h\right)}^{2}}{a^{2}}+\frac{{\left(z-k\right)}^{2}}{b^{2}}=1$$

where $\left(h,k\right)$ is the ellipse center, and a and b represent the semi-major and semi-minor axes, respectively.

#### 3.1.3. Unwanted Points Removal

Following ellipse fitting, the cross-sectional point cloud data is refined by evaluating the geometry consistency of individual points with the fitted ellipse. Specifically, points are classified based on their distances to the ellipse: those within two standard deviations of mean distance are retained as part of the tunnel lining, while outliers typically corresponding to noise, track points, bolt holes, or auxiliary facilities are flagged for removal. This statistical thresholding utilizes the normal distribution of lining points to distinguish structural components from non-lining features. This refinement process is iterative: after removing outliers, the ellipse is refitted to the remaining points, and the distance-based filtering is reapplied until the point set stabilizes (i.e., no further significant changes occur in the fitted ellipse parameters). This iterative approach ensures that the final point cloud accurately represents the geometric integrity of tunnel lining, effectively suppressing interference from ancillary structures. By progressively isolating the lining points, this step lays a robust foundation for subsequent leakage detection by minimizing false positives from irrelevant features.

### 3.2. Neighborhood Selection and Feature Generation

After preprocessing the tunnel point cloud data and isolating the tunnel lining points, the next critical step involves feature selection and design. This process begins with defining the local neighborhood around each point, which is essential for describing the 3D structure through geometric and intensity-based features. Neighborhood selection establishes the local spatial context for each point, with its effectiveness directly influencing feature relevance and accuracy. Common approaches include k-neighborhood, spherical neighborhood, and cylindrical neighborhood, as described in [32]. In this research, k-nearest neighbors (k-NN) are employed to define the neighborhood for each point. Based on these neighborhoods, five discriminative features are derived, including smoothed intensity, distance to the fitted ellipse, anisotropy, isotropy, and sphericity. The descriptions of these features are summarized as follows:

Intensity feature. Leakage points typically exhibit lower intensity values than the tunnel lining. This difference enables distinguishing leakage regions from the intact tunnel lining. However, scanning occlusions may introduce noisy intensity values, leading to potential misclassification of some lining points as leakage. To address this, the smoothed intensity is computed by averaging the intensity values within the k-neighborhood using Equation (4).

(4) $$Smoothed\;instensity=\sum_{i=1}^{k}intensity_{i}/k$$

where k Intensity feature. Leakage points typically exhibit lower intensity values than the tunnel lining. This difference enables distinguishing leakage regions from the intact tunnel lining. However, scanning occlusions may introduce noisy intensity values, leading to potential misclassification of some lining points as leakage. To address this, the smoothed intensity is computed by averaging the intensity values within the k-neighborhood using Equation (4).

Geometric features. Geometric features capture spatial structural variations and include four geometric features:

(a) Distance to the fitted ellipse: it measures the 2D distance from each point in a slice to the fitted ellipse. Leakage points near circumferential joints often protrude slightly, resulting in larger distances compared to lining. The distance is calculated using Equation (5):

(5) $$d=|\frac{{\left(x-h\right)}^{2}}{a^{2}}+\frac{{\left(z-k\right)}^{2}}{b^{2}}-1|$$

where |⋅| represents the absolute value.

(b) Anisotropy: it reflects the variation in data distribution across different directions. Leakage regions exhibit irregular shapes, leading to high anisotropy in the local point distribution.

(c) Isotropy: it reflects the uniformity of data across all directions. In contrast to anisotropy, leakage exhibits lower isotropy, whereas the tunnel lining demonstrates higher isotropy.

(d) Sphericity: it quantifies the similarity of local point clusters to a sphere. Leakage points with irregular distributions have lower sphericity, while tunnel lining points exhibit higher sphericity. More explanation about anisotropy, isotropy and sphericity can be seen in [32].

To ensure cross-scale and cross-dataset comparability, all geometric features are normalized using Equation (6). In such a way, feature values remain within [0, 1].

(6) $$F’=(F-F_{min})/(F_{max}-F_{min})$$

where $F_{min}$ represents the minimum values of features, and $F_{max}$ are the maximum values of features.

### 3.3. Optimal Neighborhood Scale Determination

The selection of an optimal neighborhood scale is critical for accurate feature extraction and subsequent leakage detection. Traditional scale selection often relies on heuristic knowledge or empirical rules. However, such methods may lack generalizability across diverse datasets. To address this, various data-driven approaches have been proposed, such as analyzing local surface variations [33], iterating geometric features for scale determination [34], using eigenentropy for scale selection [35], and aligning scales with object-specific recall thresholds [32]. These methods ensure scale selection is aligned with the dataset characteristics and analytical objectives.

In this study, the focus is on optimizing the F_1-score_, which is a harmonic mean of precision and recall, to balance extraction completeness and accuracy of leakage detection. The determination of optimal neighborhood scale follows a systematic evaluation approach. For each candidate neighborhood scale (5, 10, 20, 40, 60, 80, and 100), the method generates distinct training and validation datasets using the corresponding scale parameters. Each classifier undergoes independent training on its scale-specific training dataset, with subsequent performance evaluation conducted on the corresponding validation dataset. The scale yielding the highest F_1-score_ is selected as the optimal value. This systematic approach guarantees that the selected scale maximizes segmentation accuracy while minimizing the influence of noise and outliers. By systematically testing a broad range of scales, this method provides a robust framework for determining the optimal scale, enhancing both the precision and reliability of tunnel leakage detection.

### 3.4. Classifier Selection

XGBoost (Extreme Gradient Boosting) is a state-of-the-art machine learning algorithm renowned for its exceptional efficiency and performance in classification tasks [36]. It operates within a gradient boosting framework, iteratively constructing an ensemble of weak learners where each new learner focuses on correcting the residuals from previous predictions. This iterative process unfolds as follows: (1) an initial prediction is made for all data instances; (2) a new decision tree is trained to model the errors, or residuals, of the current ensemble; (3) this new tree is added to the ensemble, scaled by a learning rate to control its impact; and (4) these steps are repeated until the model’s performance no longer improves. Mathematically, the ensemble model at iteration t us updated as:

(7) $${\overset{\wedge }{y}i}^{\left(t\right)}(x)={\overset{\wedge }{y}i}^{\left(t-1\right)}(x)+\eta \cdot f_{t}(x_{i})$$

where ${\overset{\wedge }{y}i}^{\left(t\right)}(x)$ denotes the ensemble model at iteration, t, ${\overset{\wedge }{y}i}^{\left(t-1\right)}(x)$ represents the model from the previous iteration, $f_{t}(x_{i})$ is the newly trained weak learner based on prediction residuals, and η denotes the learning rate and controls the contribution of $f_{t}(x_{i})$.

The strength of XGBoost lies in its ability to combine multiple weak learners into a robust ensemble while mitigating overfitting through regularization techniques. Additionally, its parallel processing capability via multi-threading significantly accelerates training on large datasets, and its handling of sparse or missing data further enhances adaptability to real-world scenarios. While other modern gradient boosting algorithms such as LightGBM and CatBoost exist, XGBoost was specifically chosen for this study due to its optimal balance of predictive performance, robustness, and, most critically, its powerful and well-established tools for feature interpretability. These features make XGBoost particularly suitable for processing complex 3D point cloud data with high dimensionality and structural variability.

In this study, XGBoost is employed to utilize the five-dimensional feature space (smoothed intensity and four geometric features) derived from optimal neighborhood scales (as detailed in Section 3.3). By integrating both intensity and geometric deviations, the classifier captures subtle distinctions between leakage and non-leakage points. By systematically optimizing the neighborhood scale and feature normalization (Section 3.2), XGBoost effectively models the non-linear relationships within the multi-feature dataset, ensuring accurate leakage detection with balanced precision and recall. This combination of data-driven feature engineering and a robust ensemble learning approach positions XGBoost as an ideal choice for addressing the complexity of tunnel point cloud analysis.

### 3.5. Performance Evaluation Metrics

The performance of the proposed methodology was quantitatively evaluated using the standard classification metrics of precision, recall, and F_1-score_ [32,37]. The calculation of these metrics relies on a comparison against a reliable ground truth, which for this study was established through a rigorous manual segmentation process. This procedure involved independent labeling of the point clouds by several researchers using CloudCompare v2.14 software, with on-site photographs providing an essential visual reference to ensure accuracy. Following the initial labeling, a joint review was conducted to resolve any discrepancies until a unanimous consensus was reached for the final reference data. These metrics are derived from the four components of the confusion matrix, which compares the model’s predictions against this established ground truth: true positives (TP), false positives (FP), true negatives (TN), and false negatives (FN). Precision, which measures the exactness of the classifier, is calculated using Equation (8); recall, representing a measure of completeness, is calculated using Equation (9); and the F_1-score_, which provides the harmonic mean of precision and recall, is determined via Equation (10).

(8) $$Precision=\frac{TP}{TP+FP}$$

(9) $$Recall=\frac{TP}{TP+FN}$$

(10) $$F_{1\text{-}score}=\frac{2\times Precision\times Recall}{Precision+Recall}$$

## 4. Experimental Results and Discussions

### 4.1. Experimental Data Collection

**Figure 4:**
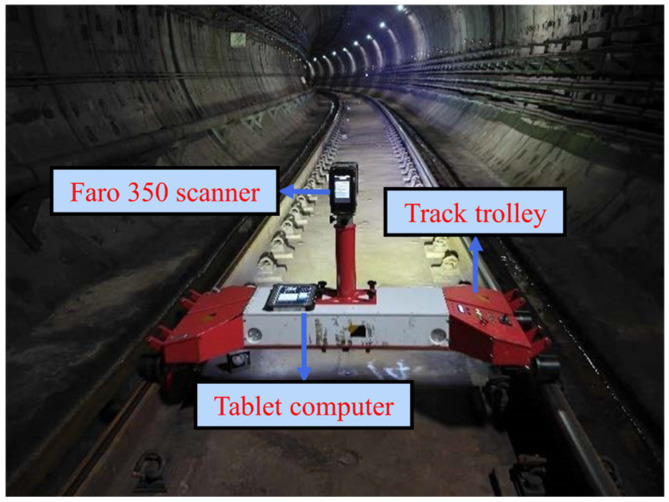
TLSD system for tunnel point cloud data collection.

This study utilized point cloud data from two shield tunnel sections from Nanjing Metro Line #2 and Line #10, spanning 1400 m (Dataset 1) and 1600 m (Dataset 2), respectively. Both tunnels are constructed from standard precast concrete segments, which is a typical design for such infrastructure. The tunnel point cloud data were collected using the Track Laser Scanning and Detection (TLSD) system, as illustrated in Figure 4. The TLSD system is composed of three primary components. The first is the Faro Focus 350 laser scanner, a high-performance instrument characterized by a ranging accuracy of ±1 mm, a scanning speed of up to 976,000 points per second, and a maximum scanning range of 350 m. Its compact dimensions (230 × 183 × 103 mm) and low weight (4.2 kg including battery) make it highly suitable for mobile applications within confined tunnel spaces. The second component is a track-mounted trolley, which provides stable driving power and mobility for the system along the tunnel. The third is an industrial tablet computer, which facilitates real-time monitoring and data processing during acquisition.

The data acquisition procedure was systematically executed. First, key operational parameters were established, including a point cloud resolution of 3.1 mm at a 10 m distance, a scanning frequency of 50 Hz, and a constant trolley speed of 0.5 m/s. The scanner then performed a continuous two-dimensional spiral scan of the tunnel cross-section, recording the scan distance, incidence angle, and timestamp for each measured point. The scanning distance is inherently variable, with its range determined by the standard 5.5 m inner diameter of the tunnels and the central position of the track-mounted system.

Finally, the system synthesized these raw measurements with the trolley’s velocity data to compute the precise three-dimensional coordinates for the entire point cloud.

### 4.2. Experimental Setting and Parameter Configuration

In this study, the proposed approach was implemented on a workstation equipped with an Intel Core i7-12700KF CPU (manufactured by Intel Corporation, Santa Clara, CA, USA), an NVIDIA GeForce RTX 3080Ti GPU (manufactured by NVIDIA Corporation, Santa Clara, CA, USA), and 64 GB of RAM (sourced from Kingston Technology, Fountain Valley, CA, USA), running Windows 10 with Python 3.8. XGBoost was adopted for leakage detection. Several key hyperparameters were configured as follows: the number of decision trees, representing the number of boosting iterations, was set to 100; the learning rate was set to 0.3, regulating the update step in each iteration; and the maximum tree depth was specified as 6, which denotes the maximum splitting depth for each node. This setup strikes a balance between computational overhead and detection accuracy.

### 4.3. Data Preprocessing Results

**Figure 5:**
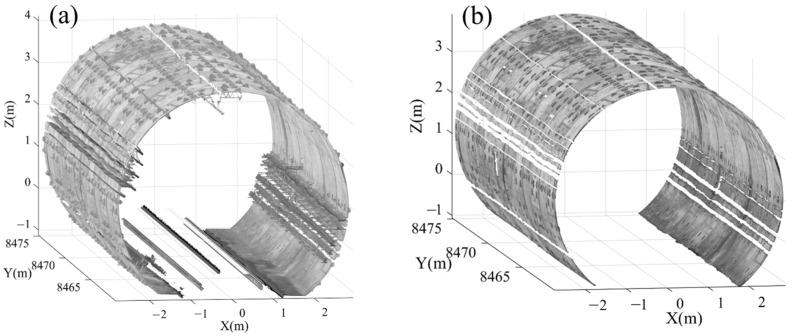
Example results of point cloud data preprocessing. (**a**) Raw point cloud. (**b**) Denoised point cloud.

Following the acquisition of point cloud data, preprocessing was conducted as detailed in Section 3.1. Figure 5 displays the denoising results for a segment of Dataset 1. As illustrated in Figure 5, the preprocessing effectively removed points associated with rails, auxiliary facilities, ground, and bolt holes. These results demonstrate that the capability of the method to minimize point cloud noise and mitigate interference in leakage detection, thereby establishing a robust foundation for the subsequent detection process.

The preprocessed data now exclusively comprises tunnel linings and leakage points. The subsequent steps involve sample selection for model training and testing. Due to the relatively small proportion of leakage points compared to tunnel lining points, sections with a higher incidence of leakage were chosen for training. Table 2 summarizes the key characteristics of the selected samples, including the leakage type, point density, tunnel length, number of points, and leakage–lining point count ratio.

### 4.4. Optimal Neighborhood Scale Results and Analysis

The optimal neighborhood scale was determined by maximizing the F_1-score_ across various values of k. Table 3 presents the recall, precision, and F_1-score_ for various k-values across both datasets. For Dataset 1, the highest F_1-score_ of 91.18% was achieved with a k-value of 5. For dataset 2, the highest F_1-score_ of 97.84% was obtained with a k-value of 10. Therefore, the optimal neighborhood scales for the two datasets are 5 and 10, respectively. The variation in optimal scales between the two datasets may be attributed to the larger minimum point spacing in Dataset 2, which requires a larger scale to capture a sufficient number of neighborhood points.

### 4.5. Leakage Detection Results

**Figure 6:**
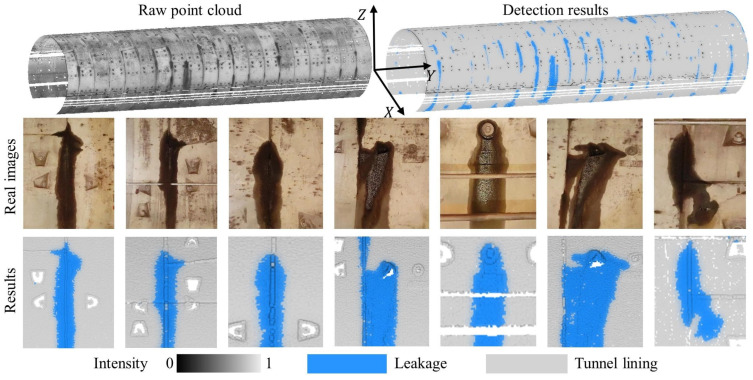
Example results of leakage detection for Dataset 1.

**Figure 7:**
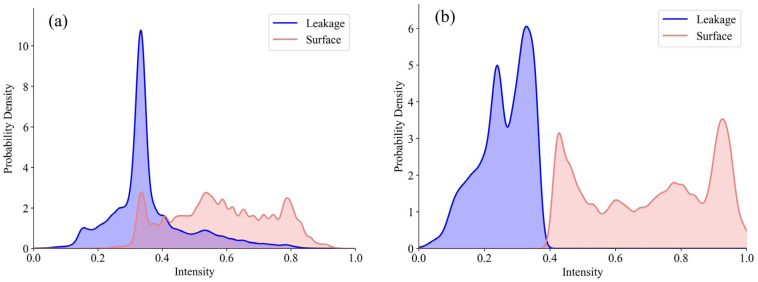
Intensity probability density for both datasets. (**a**) Dataset 1. (**b**) Dataset 2.

To investigate the contribution of intensity features and geometric features on leakage detection accuracy, ablation experiments were conducted. Figure 6 demonstrates that the proposed method accurately detects and effectively segments leakages in 3D space, with the segmented leakage shapes closely matching their corresponding ground truth representations. Table 4 further presents the recall, precision, and F_1-score_ for different feature sets across both datasets. For the two datasets, classification using only intensity features resulted in a precision of 87.17% and 95.56% for leakage detection, respectively. This indicates that intensity features are effective in distinguishing leakages from non-leakages. In Dataset 2, the recall for leakage reached 98.27%, demonstrating the efficacy of intensity features in extracting leakages. However, the lower recall for leakage in Dataset 1 may be attributed to a significant overlap in intensity distributions between leakage and tunnel lining, which diminishes the effectiveness of intensity features for leakage, as illustrated in Figure 7.

### 4.6. Comparison with Other Frequently Used Classifiers

**Figure 8:**
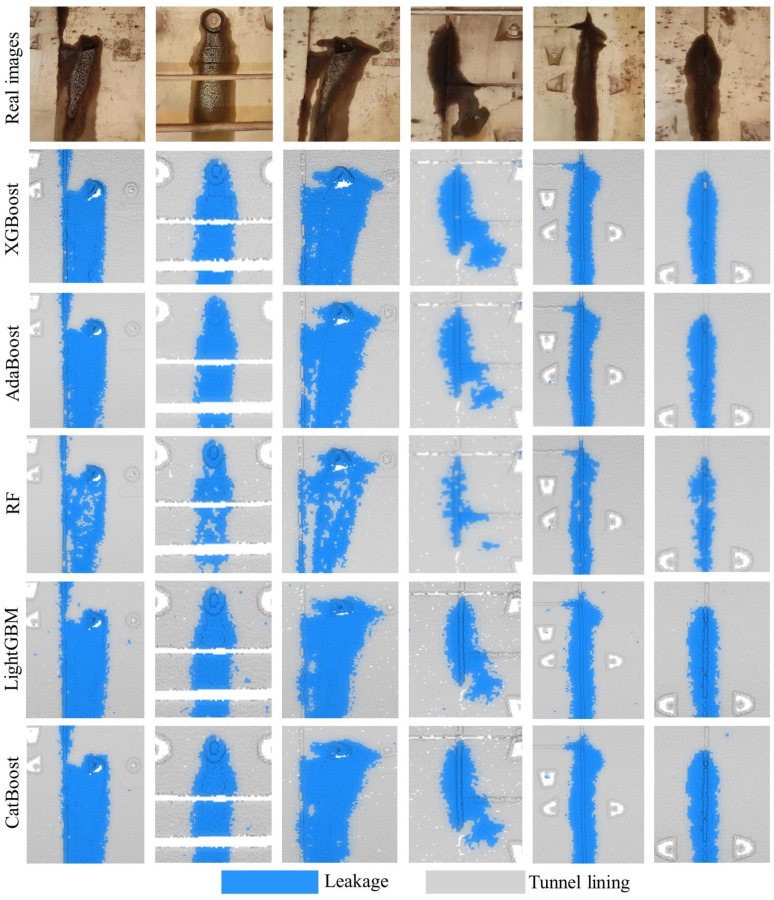
Example results of leakage detection from different classifiers presented in a 2D side view for Dataset 1.

To validate the accuracy and efficiency of the selected classifier, XGBoost was benchmarked against four other high-performance models commonly employed for classification tasks: AdaBoost, Random Forest (RF), LightGBM, and CatBoost. The comparative performance was evaluated based on the metrics of precision, recall, and F_1-score_, as presented in Table 5, while the computational runtime for each classifier is detailed in Table 6. Figure 8 provides a qualitative illustration of the leakage detection results produced by the different classifiers on Dataset 1.

As shown in Table 4, XGBoost achieves the highest recall for leakage detection among all classifiers in both datasets, a result which corresponds with the visualizations in Figure 8. This indicates that XGBoost is particularly effective in identifying potential leakage areas. While it exhibits slightly lower precision compared to some classifiers, its superior recall culminates in the highest overall F_1-score_ for both datasets. This demonstrates that XGBoost most effectively and reliably segments leakage from the tunnel lining among the models tested.

The analysis of computational efficiency, presented in Table 5, reveals a more nuanced landscape. For inference, XGBoost demonstrates exceptional speed, with a testing time of only 0.03 min on Dataset 1 and a near-instantaneous 0.001 min on Dataset 2. In terms of training, while LightGBM exhibits the fastest performance on Dataset 1 (6.13 m), the training time for XGBoost (9.43 m) remains highly competitive and is orders of magnitude faster than traditional models like AdaBoost (5308.25 m) and Random Forest (956.40 m). This efficiency can be attributed to the optimized gradient boosting framework of XGBoost, which supports parallel and distributed computing, facilitating rapid model convergence. When considering both its state-of-the-art accuracy and its excellent computational performance, particularly its rapid inference speed, XGBoost emerges as the most balanced and practical choice for this application.

### 4.7. Comparison with Different Methods

To validate the superiority of the proposed method, its performance was benchmarked against three prominent deep learning models: PointNet, PointNet++, and DGCNN. All models were evaluated on Dataset 1 using an identical input data format of [x, y, z, intensity]. The comprehensive results, which include both accuracy and computational runtime, are presented in Table 7.

In terms of segmentation accuracy, the proposed method significantly outperforms the deep learning models across all key metrics, including precision, recall, and F_1-score_. This enhanced performance can be attributed to the method’s ability to effectively capture subtle geometric features through neighborhood analysis and its robust handling of intensity variations, which are crucial for distinguishing leakage from the tunnel lining. Regarding computational efficiency, the proposed method demonstrates a pronounced advantage, particularly in the inference phase. As detailed in Table 6, the test time is orders of magnitude lower: only 0.03 min compared to the 9 to 45 min required by the deep learning architectures. It is also noteworthy that the total training time for the proposed method (2 h 9 m) encompasses both feature extraction and model training. The XGBoost model training itself was remarkably swift, requiring only 9.43 min, as shown in Table 5, with the remainder of the time dedicated to the one-time feature extraction process. This efficiency stems from avoiding the computationally intensive, end-to-end feature learning inherent in deep learning networks.

**Figure 9:**
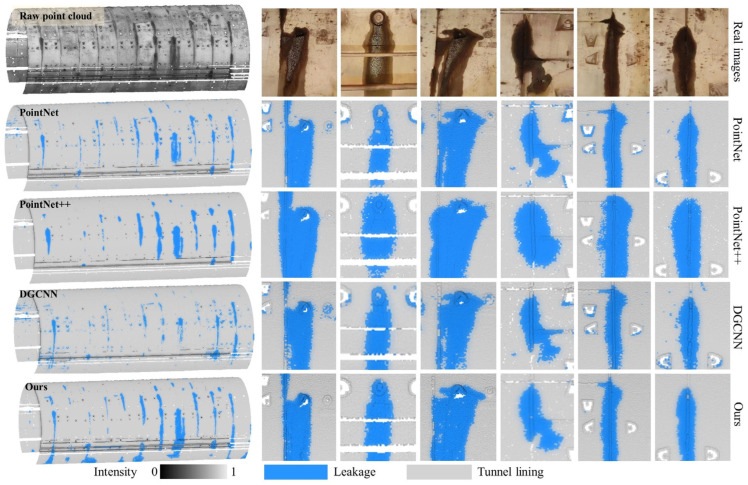
Comparative results of different methods for Dataset 1.

These quantitative findings are further supported by the qualitative results illustrated in Figure 9. While all three deep learning models effectively detect leakages, they exhibit certain limitations; PointNet++ is prone to over-segmentation, and both DGCNN and PointNet misclassify some tunnel lining points as leakage. In contrast, the proposed method successfully discriminates between leakages and tunnel lining, achieving a more precise segmentation that aligns closely with the ground truth shapes.

Collectively, these results confirm that the proposed method provides a compelling balance of high accuracy and superior computational efficiency, making it a robust and practical solution for tunnel leakage detection.

### 4.8. Generalizability of the Proposed Method

To evaluate the generalization performance of the proposed method, the model trained on Dataset 1 was tested on four additional heterogeneous datasets. These datasets were captured using various scanners, including Leica, Faro, and Z + F, and feature a range of point densities, as detailed in Table 8. The method demonstrated strong and consistent performance on Datasets 3, 4, and 5, all of which represent shield tunnels. The F_1-score_ for these datasets were 84.46%, 84.66%, and 96.06%, respectively. This consistent performance across diverse shield tunnel data confirms the robust generalization capability of the proposed method.

In contrast, a notable performance difference was observed for Dataset 6. While the model maintained excellent recall (99.88%), its precision was significantly lower at 73.20%. This performance drop is attributable to the fundamental design of the proposed method, which is specifically optimized for circular shield tunnels. The approach enhances detection accuracy by identifying the distinct geometric characteristics of ring seams and by utilizing the “Distance to ellipse” feature. The horseshoe-shaped cross-section of Dataset 6, however, violates these core geometric assumptions. This structural difference not only renders the “Distance to ellipse” feature inapplicable, but also means the tunnel lacks the regular, pronounced ring seams that the classifier uses as crucial contextual cues. The resulting drop in precision is therefore a logical consequence of applying a specialized model to a fundamentally different tunnel structure.

### 4.9. Importance of Geometric Features

**Figure 10:**
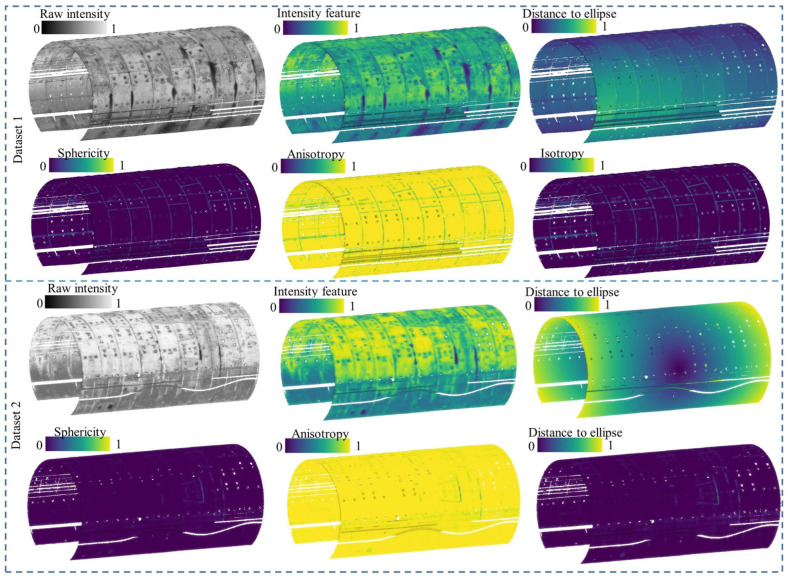
Graphical illustration for the geometric features of both datasets.

To validate the significance of geometric features, ablation studies were conducted. As presented in Table 9, the inclusion of geometric features yielded significant improvements for Dataset 1, with precision, recall, and F_1-score_ increasing by 12.56%, 14.28%, and 13.36%, respectively. In contrast, their impact on Dataset 2 was minimal, where recall and F_1-score_ rose by only 0.95% and 0.46%, respectively. The observed performance discrepancy can be primarily attributed to variations in tunnel structural conditions and the resulting impacts of preprocessing. In practical engineering scenarios, leakages frequently occur at structural discontinuities, such as ring seams and bolt holes, which present distinct geometric characteristics compared to the relatively uniform tunnel lining. As illustrated by Table 2 and the “Distance to ellipse” feature in Figure 10, Dataset 2 exhibited more significant cross-sectional deformation than Dataset 1. This condition necessitated a more aggressive denoising procedure, which inevitably removed numerous points corresponding to ring seams and bolt holes. The loss of critical geometric information hindered the effective identification of leakage features.

**Figure 11:**
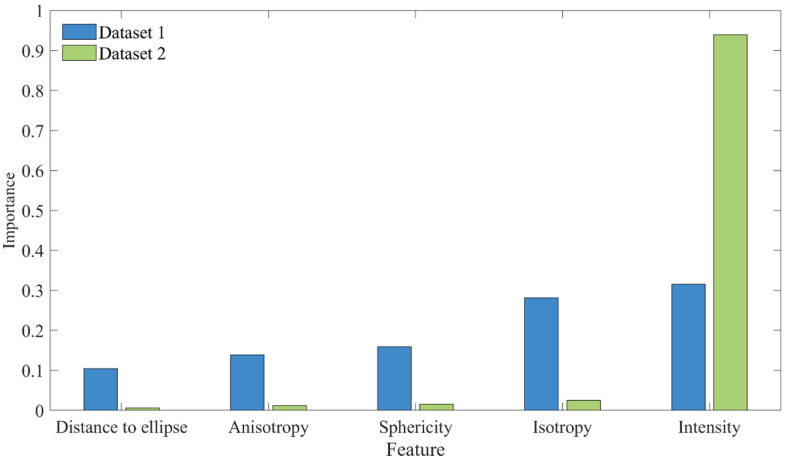
Graphical illustration for the feature importance of both datasets.

The feature importance analysis, illustrated in Figure 11, further corroborates this explanation. Geometric features demonstrated greater importance in Dataset 1, which, as shown in Figure 10, retained more points associated with seams and bolt holes. Additionally, intensity features were overwhelmingly dominant in Dataset 2. This aligns with the observations in Section 4.5, where the intensity values for leakage and non-leakage areas in Dataset 2 show minimal overlap, making intensity a more discriminative feature for leakage detection.

While the proposed method demonstrates promising accuracy, two primary limitations were identified that also suggest clear directions for future research. First, a significant overlap in intensity distributions can occasionally occur between leakage areas and the tunnel lining, which diminishes the discriminative power of the intensity feature. A potential mitigation strategy involves developing an effective intensity correction technique to reduce this overlap. Second, for tunnels with severe cross-sectional deformation, the necessarily aggressive denoising process can inadvertently remove the subtle geometric features that are critical for detection. Therefore, future work could focus on designing a deformation-aware denoising algorithm. Such a method would be tailored to preserve the geometric integrity of structural features like ring seams, thereby enhancing the robustness of the feature extraction process.

## 5. Conclusions

This study presents a novel framework that integrates both geometric and intensity features for robust leakage detection. A pivotal advancement of this framework is the synergistic use of engineered geometric features with a highly efficient XGBoost classifier. The method employs robust ellipse fitting to handle structural noise and then generates key geometric descriptors from the point cloud. These features, combined with intensity data, create a multi-dimensional representation of each point. The XGBoost classifier is effectively utilized within this multi-feature space to accurately distinguish leakage from the tunnel lining. Extensive experiments demonstrate the superior performance of this approach, which achieves F_1-scores_ of 91.18% and 97.84% on two datasets. A direct comparison with other prominent classifiers, including RF, AdaBoost, CatBoost, and LightGBM, demonstrated that XGBoost provides the best balance of accuracy and efficiency. Furthermore, the proposed method surpasses state-of-the-art deep learning models, including PointNet, PointNet++, and DGCNN, in both segmentation accuracy and computational speed. Additionally, robust performance across four heterogeneous datasets validates the strong cross-device and cross-site generalization capabilities of the framework, confirming the efficacy and practicality of the proposed method.

## Figures and Tables

**Table 1:** Comparison of representative leakage detection methods. Note: E3NCA denotes the Efficient 3-Normalized Conv1Ds of Attention module; Soft-NMS denotes the Soft Non-Maximum Suppression; FPN denotes Feature Pyramid Network; IoU measures the overlap between the predicted leakage area and the ground truth; mIoU is the average IoU across all classes. AP is the average value of precision; AP50 and AP75 are the AP calculated at an IoU threshold of 0.50 and 0.75, respectively; mAP is the average AP over all classes; NA indicates that the data is not available.

Approach	Source	Structure	Equipment	Used Data Type for Detection	Detection Techniques	Detection Accuracy	Limitations
Intensity threshold-based segmentation	Huang, et al. [7]	Shield	MTI-100 system (Line array camera)	Image	Otsu method	NA	(1) Sensitive to lighting conditions and object occlusion (2) Sensitive to varying threshold (3) Failure to visualize leakage patterns in 3D space
	Xu, et al. [15]	Rectangle	TLS system ((RIEGL VZ400i scanner)	Images projected using point clouds	Intensity threshold	NA	
Image-based supervised classification	Liu, et al. [25]	Shield	MLS system (Faro 120 & Leica P16 scanner)	Images projected using point clouds	(1) Res2Net (2) Cascade module (3) FCN	AP: 58.9% AP50: 89.7% AP75: 66.8%	(1) Sensitive to lighting conditions and object occlusion (2) Spatial information loss from 2D projection (3) Failure to visualize leakage patterns in 3D space
	Guo, et al. [1]	Shield	MLS system (Faro X120 scanner)	Images projected using point clouds	(1) YOLOv5 (2) E3NCA (3) SoftNMS	mAP: 68.9%/49.2	
	Chen, et al. [28]	Shield	NA	Images	(1) YOLOv7 (2) Attention mechanisms (3) Edge refinement	IoU: 89.97%	
	Wang, et al. [29]	Shield	Manual photography	Images	(1) DeepLabV3 (2) Channel attention	mIoU: 84.68%	
	Chen, et al. [19]	Shield	MLS system (Faro 120 scanner)/TLS system (Faro 350 scanner)	Images projected using point clouds	(1) Mask R-CNN (2) FPN (3) ResNet 50	mAP: 76.4%	

**Table 2:** Selection of training and testing samples. Length represents total mileage of tunnel. Ratio denotes the number proportion of leakage points to tunnel lining points.

Data	Mean Values of Cross-Sectional Deformation	Point Cloud Density	Leakage Type	Training Samples			Testing Samples		
				Length	Total Points	Ratio	Length	Total Points	Ratio
Dataset 1	2 mm	5865 pts/m^2^	Joint leakage	800 m	83,020,439	1:61	200 m	19,412,661	1:22
Dataset 2	4 mm	1652 pts/m^2^	Joint leakage	294 m	4,846,982	1:24	22 m	478,201	1:109

**Table 3:** The recall, precision, and F_1-score_ under different values of k (%).

k-Value	Dataset 1			Dataset 2		
	Recall	Precision	F_1-score_	Recall	Precision	F_1-score_
5	87.17	95.56	91.18	97.59	96.81	97.20
10	98.47	81.66	89.28	97.41	98.27	97.84
20	82.38	86.10	84.20	95.19	97.03	96.10
40	33.50	61.58	43.39	93.66	95.58	94.61
60	64.79	91.57	75.88	94.61	93.46	94.03
80	74.92	88.30	81.06	93.50	92.13	92.81
100	64.35	81.42	71.89	92.94	90.57	91.74

**Table 4:** The Recall, Precision, and F_1-score_ using different datasets at optimal scales (%).

Data	Structure	Source	Precision	Recall	F_1-score_
Dataset 1	Shield	Nanjing, line2	87.17	95.56	91.18
Dataset 2	Shield	Nanjing, line10	97.41	98.27	97.84

**Table 5:** The Recall, Precision, and F_1-score_ using different classifiers (%).

Classifier	Dataset 1			Dataset 2		
	Recall	Precision	F_1-score_	Recall	Precision	F_1-score_
XGBoost	87.17	95.56	91.18	97.41	98.27	97.84
AdaBoost	75.69	97.93	85.39	91.30	98.55	94.79
RF	51.89	96.99	67.61	88.85	98.59	93.46
LightGBM	84.20	84.51	84.35	87.86	93.80	90.73
CatBoost	84.42	89.22	86.75	94.21	94.47	94.34

**Table 6:** The execution time of different classifiers. Note: unit is minute(s).

Classifier	Dataset 1		Dataset 2	
	Training	Testing	Training	Testing
XGBoost	9.43	0.03	0.14	0.001
AdaBoost	5308.25	0.58	6.93	0.02
RF	956.40	0.15	0.93	0.006
LightGBM	6.13	0.08	0.05	0.001
CatBoost	90.78	0.10	2.76	0.001

**Table 7:** Comparison with different methods (%). Note: h represents hour(s). m denotes minute(s).

Method	Precision	Recall	F_1-score_	Train Time	Test Time	Total Time
PointNet	70.70	53.20	60.70	2 h 14 m	45 m	2 h 59 m
PointNet++	74.62	52.95	61.94	4 h 8 m	42 m	4 h 50 m
DGCNN	54.78	76.11	63.71	3 h 50 m	9 m	3 h 59 m
Ours	95.56	87.17	91.18	2 h 9 m	0.03 m	2 h 9.03 m

**Table 8:** The recall, precision, and F_1-score_ using heterogeneous datasets (%).

Data	Structure	Source	Scanner Type	Point Cloud Density	Precision	Recall	F_1-score_
Dataset 3	Shield	Nanjing, line 3	Z + F	1685 pts/m^2^	81.64	87.49	84.46
Dataset 4	Shield	Wuxi, line 2	Faro	3094 pts/m^2^	83.65	85.71	84.66
Dataset 5	Shield	Hangzhou, line 2	Leica	1677 pts/m^2^	94.28	97.90	96.06
Dataset 6	Horseshoe	Nanjing	Leica	2086 pts/m^2^	73.20	99.88	84.48

**Table 9:** The recall, precision, and F_1-score_ using different feature sets (%).

Data	Feature Sets	Precision	Recall	F_1-score_
Dataset 1	Intensity feature	74.61	81.28	77.82
	Intensity feature+ Geometric features	87.17	95.56	91.18
Dataset 2	Intensity feature	97.43	97.32	97.38
	Intensity feature + Geometric features	97.41	98.27	97.84

## Data Availability

The datasets presented in this article are not readily available because of project privacy restrictions.
